# Cross-talk between HIF and p53 as mediators of molecular responses to physiological and genotoxic stresses

**DOI:** 10.1186/1476-4598-12-93

**Published:** 2013-08-14

**Authors:** Joanna Obacz, Silvia Pastorekova, Borek Vojtesek, Roman Hrstka

**Affiliations:** 1 Masaryk Memorial Cancer Institute, Regional Centre for Applied Molecular Oncology, Zluty kopec 7, 65653 Brno, Czech Republic; 2 Department of Molecular Medicine, Institute of Virology, Slovak Academy of Sciences, Dubravska cesta 9, 84505 Bratislava, Slovak Republic

**Keywords:** p53, HIF-1, Hypoxia, DNA damage, Cancer

## Abstract

Abnormal rates of growth together with metastatic potential and lack of susceptibility to cellular signals leading to apoptosis are widely investigated characteristics of tumors that develop via genetic or epigenetic mechanisms. Moreover, in the growing tumor, cells are exposed to insufficient nutrient supply, low oxygen availability (hypoxia) and/or reactive oxygen species. These physiological stresses force them to switch into more adaptable and aggressive phenotypes. This paper summarizes the role of two key mediators of cellular stress responses, namely p53 and HIF, which significantly affect cancer progression and compromise treatment outcomes. Furthermore, it describes cross-talk between these factors.

## HIF-mediated responses to hypoxia

Important consequences of rapid tumor growth include poor vascularization and insufficient oxygen delivery that together lead to formation of hypoxic (poorly oxygenated) areas [1]. Adaptation to hypoxia is facilitated by the activation of transcriptional machinery, in which hypoxia inducible factor (HIF) plays a pivotal role. HIF is a heterodimeric transcription factor composed of an oxygen-dependent α-subunit and constitutively expressed β-subunit. Regulation of the α-subunit is driven by enzymes of the prolyl hydroxylase family (PHDs) and by the factor inhibiting HIF (FIH) [2,3]. Under normoxia, PHDs hydroxylate prolines at positions 564 and 402 (in HIF-1α isoform) and FIH hydroxylates asparagine at position 803 [3]. Hydroxylation of prolines is required for recognition of HIF-1α by the ubiquitin ligase complex via von Hippel-Lindau (pVHL) tumor suppressor protein, which in consequence leads to HIF-1α ubiquitination followed by its proteasomal degradation [2]. Simultaneously, FIH prevents interaction between HIF-1α and the transcriptional co-activator, p300. Although there are three isoforms of the α-subunit: HIF-1α, HIF-2α and HIF-3α, most attention is drawn to HIF-1α and HIF-2α. These subunits contain similar oxygen-dependent degradation domains, but play different roles in hypoxic tumor growth and progression (for extended review see Keith et al.) [4]. Whereas HIF-1 mediates acute responses to hypoxia, HIF-2 is more involved in adaptation to chronic hypoxia and is functionally implicated in tumor progression [5].

In situations of insufficient oxygen levels, PHDs and FIH remain inactive, while HIF-1α is no longer hydroxylated and escapes recognition by pVHL. This results in its stabilization, accumulation and translocation to the nucleus, where it interacts with a β-subunit leading to creation of an active heterodimeric form of the transcription factor. This heterodimer binds to specific cis-acting hypoxia responsive elements (HREs) in the promoters of target genes [6].

Several recent reports point out novel molecular mechanisms that affect HIF-1α levels in normoxia. An inhibitor of Janus Activated Kinase (JAK2), AG490, prevents HIF-1α hydroxylation and thus interferes with VHL-mediated degradation resulting in increased HIF-1α protein half-life [7]. Another mechanism by which HIF-1α can be rescued from degradation is via interaction with ubiquitin-specific protease 19 (USP19) [8]. Epigenetic mechanisms such as histone methylation can also be involved in HIF-1α regulation, which was studied in clear cell renal cell carcinoma (ccRCC) [9]. Moreover, HIF-1 activity is phosphorylation-dependent and thus requires engagement of signaling such as mitogen-activated protein kinase (MAPK), PI3K/Akt and mammalian target of rapamycin (mTOR), amongst others (see review by Dimova et al.) [10].

HIF-induced cascades of events allow cells to survive and overcome unfavorable conditions during hypoxia by transcriptional reprogramming that leads to modulated proliferation, angiogenesis, cell metabolism and many other features of tumor phenotype. One of the prominent HIF-1 downstream genes involved in this process is the gene coding for carbonic anhydrase IX (CA IX). CA IX is a member of the family of zinc metalloenzymes involved in regulation of cellular pH by reversible conversion of CO_2_ to bicarbonate and proton [11,12]. Its activity is regulated by hypoxia through protein kinase A and leads to acidosis of the tumor milieu, which is known to be one of the hallmarks of solid tumors [13,14]. CA IX also promotes tumor cell growth and survival and helps to eliminate the surplus of intracellular acids generated through oncogenic metabolism [15,16]. Moreover, it facilitates migration and invasiveness of tumor cells and thereby supports tumor progression [17].

To satisfy the need for nutrients, tumor cells are forced to create an extensive net of new vessels through increased expression of pro-angiogenic molecules, including vascular endothelial growth factor (VEGF), which is also a well-known HIF target gene [18,19]. Additionally, VEGF can promote both angiogenesis and metastasis via up-regulation of matrix metalloproteinase 28 and matrix metalloproteinase 14 [20].

Due to lack of oxygen, a key factor for respiration, hypoxia is also known to induce a shift to glycolytic metabolism [21]. HIF-1 plays a growth factor-dependent role in the regulation of glycolysis in hematopoietic cells even in the absence of hypoxia [22] and reduces mitochondrial respiration in RCC lacking VHL [23]. HIF was also shown to be responsible for expression of specific isoforms of glycolytic enzymes and transporters via alternative splicing [24].

There are many other molecular targets of HIF that execute multiple adaptive responses to hypoxia depending on the cell type and physiological context as described elsewhere [25,26].

## p53-mediated responses to genotoxic stress

Tumor suppressor p53, which shows many similarities to HIF-1 in terms of protein control by degradation, is predominantly involved in adaptation of cells to genotoxic stresses. p53 is a well-characterized transcription factor that plays a crucial role in responses to DNA damage, aberrant cell cycle control, apoptosis, and senescence [27-29]. Comparably to HIF-1α, the basal level of wild-type p53 is kept low due to murine double minute 2 (MDM2)-dependent ubiquitination [30]. In response to DNA damage p53 is stabilized and phosphorylated by ataxia telangiectasia mutated (ATM) protein, which leads to its activation and binding to the regulatory region of target genes [31,32]. Moreover, p53 can be regulated through methylation caused by MDM2-dependent recruitment of methyltransferases [32]. In contrast, MDM2 can also act as a p53 inducer. This is mediated through the interaction of p53 mRNA region containing the MDM2-binding site with the RING domain of MDM2, which impairs the E3 ligase activity of MDM2 and promotes p53 mRNA translation [33]. This interaction depends on ATM-mediated phosphorylation of MDM2 at Ser395 [34]. Finally, activated p53 can then start the machinery leading either to cell cycle arrest and DNA repair or to apoptosis. For example, p53-dependent upregulation of genes involved in inhibition of IGF-1/AKT and mTOR pathways prevents cell growth and division [29,35,36]. On the other hand, inhibition of DNA damage-activated kinases leads to switch of the p53-dependent growth arrest to apoptosis [37].

ATF3 gene, a downstream target of p53, encodes a transcription factor involved in adaptation to hypoxia, ER stress, oxidative stress and genotoxic stress [38]. ATF3 acts both as an effector of p53-mediated cell death and a regulator of p53 signaling. A recent report indicates that ATF3 has opposing effects on apoptotic transcriptome in stress response and in cancer, where it was found to be over-expressed [39]. Zhang and colleagues [40] developed a four-module model to investigate p53 dynamics and the DNA damage response. They found that primary modifications such as phosphorylation at Ser-15 and Ser-20 cause cell cycle arrest, whereas further modifications such as phosphorylation at Ser-46 fully activate p53 which can then induce apoptosis. This report more clearly elucidates how p53 converts between the cell cycle arrester and the killer, which was previously shown to be controlled by Wip1 (wild-type p53-induced phosphatase 1) [41].

p53 does not only act as a transcriptional factor in the nucleus, but also can move to the mitochondria where it induces permeabilization of the mitochondrial outer membrane consequently releasing pro-apoptotic factors [28]. Suppression of autophagy via inhibition of AMP-dependent kinase and/or activation of mTOR is another cytoplasmic p53 function [42]. For the extensive insight into the cytoplasmic functions of p53, see the review by Green and Kroemer [28].

p53 as a tumor suppressor plays an important role in maintaining of genome stability thus it is not surprising that is mutated in more than 50% of cancers in which its loss facilitates malignant transformation [43]. The majority of p53 mutations represent missense mutations located in the DNA-binding core domain of p53, producing a full-length protein that is incapable of binding DNA and is therefore nonfunctional as a transcriptional activator/repressor. Compared to wild-type p53, missense mutant proteins show increased stability, which is partly caused by their inability to induce MDM2 but also by the formation of complexes with HSP90 and HSP70 [44].

## Cross-talk between HIF-1 and p53

**Figure 1:**
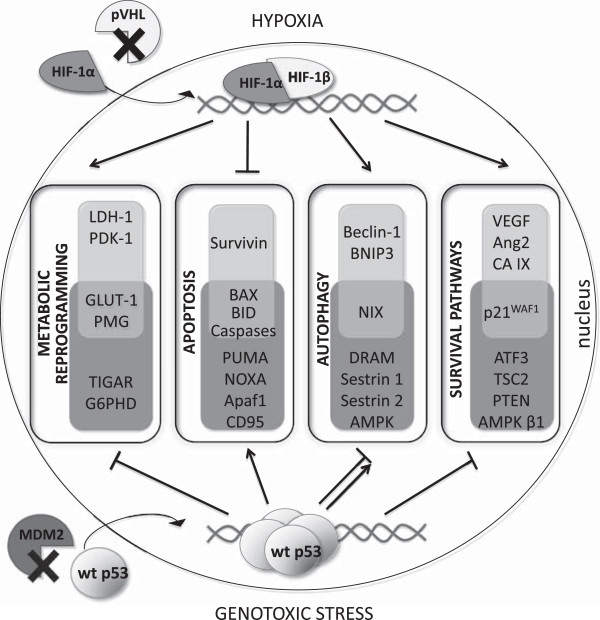
**HIF-1 and/or p53 regulated genes mediating adaptation to cellular stresses through activation of different pathways.** Upon hypoxia, the interaction between HIF-1α and von Hippel Lindau protein (pVHL) is disrupted, leading to HIF-1α translocation into nucleus, dimerization with HIF-1β subunit and formation of HIF-1 active form, which can regulate transcription of target genes . HIF-1 activates lactate dehydrogenase (LDH-A), pyruvate dehydrogenase kinase 1 (PDK1), phosphoglycerate mutase (PGM) and glucose transporter 1 (GLUT-1) to switch into more glycolytic phenotype [25]. To prevent apoptosis, it induces survivin expression [25] and downregulates BAX, BID and caspases activity [26]. HIF-1 can also induce autophagy by upregulation of beclin-1, BNIP3 and NIX [81]. Through modulating vascular endothelial growth factor (VEGF) [18], angiopioetin-2 (Ang-2) [25], carbonic anhydrase IX (CA IX) [12] and p21^WAF1^[90] expression, HIF-1 triggers activation of pro-survival pathways. Different molecular stresses (including DNA damage, hypoxia, oxidative stress), cause dissociation of p53 from murine double minute 2 (MDM2) complex, enabling its binding to regulatory elements of target genes [31]. Thereby p53 can repress glycolysis by altering expression of GLUT-1, PGM, TP53-induced glycolysis and apoptosis regulator (TIGAR) and inhibits pentose phosphate pathway by downregulating glucose-6-phosphate dehydrogenase (G6PDH) [36]. p53 regulates expression of many pro-apoptotic proteins, including PUMA, NOXA, CD95, Apaf1, BAX, BID and caspases [28]. Induction of autophagy by p53 relies on activation of damage-regulated autophagy modulator (DRAM) [83], sestrin 1, sestrin 2 and AMP-dependent kinase (AMPK) [84], but depending on cellular localization it can also inhibit this process [86]. Regulation the expression of transcription factor ATF3 enables adaptation to hypoxia, ER stress, oxidative stress and genotoxic stress [38], whereas during hypoxia induction of p21^WAF1^ causes cell cycle arrest [102]. p53 suppresses Akt-mTOR axis by transactivation of PTEN, TSC2 and AMPKβ1 [36].

In addition, p53 participates in responses to hypoxia by regulating expression of genes involved in cell cycle control. This happens via a pathway that is different than that involved in the DNA damage response [45]. There are many contradictory reports on mutual influence of p53 and hypoxic signaling. Some of them claim that hypoxia causes accumulation and increase in p53 protein level [46,47], whereas others postulate degradation-mediated decrease in p53 level [48,49] or no effect at all [50]. These intricate relations have been extensively reviewed by Sermeus and Michiels [51]. One explanation of these contradictory statements can be found in the phosphorylation status of HIF-1. It was shown that dephosphorylated HIF-1 is a major form binding to p53, precluding downregulation of p53 by MDM-2, and thus enabling it to conduct apoptosis [52]. As both p53 and HIF-1 are mediators of cell adaptation to many stresses, they are known to be involved in similar processes such as apoptosis, cell cycle control, metabolism etc. (Figure 1). Severe and/or prolonged hypoxia activates p53-dependent apoptosis, which is initiated by stabilization of 53 by HIF-1 [53]. In contrast, another report states that hypoxia causes growth arrest by decreasing p53 phosphorylation, but has no impact on either p21^WAF1^ or HIF-1 protein stabilization [54]. One of the possible explanations is that these convergences can be due to cancer cell type [55]. Opposite effects can be observed upon genotoxic stress, where wild-type 53 abrogates HIF-1 activity triggering its proteasomal degradation [56].

However, there is a line of evidence that HIF-1 can also impair p53 activity, through the downregulation of the tumor suppressor homeodomain-interacting protein kinase-2 (HIPK2) [57]. HIPK2 phosphorylates p53 at serine 46 in response to DNA damage and subsequently activates its apoptotic function [58]. Moreover, HIPK2 inhibition can result from the hypoxia-induced upregulation of MDM2 [59].

p53 can respond to DNA damage in cooperation with 70 kDa subunit of the replication protein A (RPA70). Under hypoxia, wild-type p53 undergoes a conformational change and acquires mutant conformation [60]. Furthermore, hypoxia leads to disruption of the complex between p53 and RPA70, dissociation of RPA70 and activation of RPA70-mediated nucleotide excision repair and non-homologous end-joining repair, which cause resistance to apoptosis in hypoxic cancer cells [61]. That report poses a new insight into impairment of the p53-mediated apoptosis and consequent insensitivity of cancer cells to treatment. However, it is still hard to elucidate what starts the p53 and/or HIF-1 machinery for the adaptation of cells to unfavorable conditions.

Thomas et al. [62] focused on tumor response to nitric oxide (NO) exposure and proposed that both p53 and HIF-1 are stabilized by NO in a dose- and time-dependent manner, with a higher NO concentration required for p53 stabilization. They suggested that cells localized closer to the source of NO production can undergo p53-dependent cell arrest and death, while more distant cells respond with increased HIF-1 levels. Additionally, their results indicated that HIF-1 stabilization by NO was independent of p53 status.

Altered metabolism is one of the prominent features that promote tumor survival. The first who discovered that tumors rely on anaerobic glycolysis even in the presence of sufficient oxygen and produce large amount of lactate was Otto Warburg [63]. Later this phenomenon was named after him. The consequences of this effect have been previously reviewed [64]. Another tumor characteristic is increased uptake of nutrients that as stated by Vander Heiden et al. [65] is due to oncogenic mutations mainly in Akt, Myc and Ras [66]. A multitude of mutations of genes encoding enzymes participating in glycolysis, tricarboxylic acid cycle, mitochondrial oxidative phosphorylation and other molecular pathways underlying the advantageous metabolism of cancers have been already characterized [67-71]. Comprehensive insights into this phenomenon can be found in recent works [72-75]. In this respect HIF-1 and p53 play crucial, but usually competing, roles. HIF-1 controls expression of genes encoding e.g. glucose transporters, glycolytic enzymes, lactate dehydrogenase etc. [25,76]. Interestingly, inactivating mutations in fumarate hydratase and succinate dehydrogenase cause accumulation of their substrates, which interfere with HIF-1α degradation leading to its accumulation [77]. On the other hand, loss of p53 contributes to enhancement of glucose transport and metabolism through NF-κB pathway [78]. Furthermore, it increases lactate production, diminishes oxygen consumption and enhances hypoxia-induced cell death. Disruption of p53 function reduces the expression of cytochrome c oxidase 2 (SCO2), which is necessary for the respiratory chain function [79]. This indicates that mutations in the TP53 gene contribute to Warburg effect.

In order to eliminate damaged proteins and organelles as well as to fulfill requirements for high ATP level, cancer cells utilize the machinery of autophagy, a catabolic process in which cytoplasmic cargos are embedded in double-membrane structures called autophagosomes to digest their content [80]. Among proteins involved in triggering autophagy, BCL2/adenovirus E1B 19kDa-interacting protein 3 (BNIP3), BCL2/Adenovirus E1B 19kDa Interacting Protein 3-Like (BNIP3L, NIX), together with Beclin-1 are induced under hypoxia in HIF-1-dependent manner (see review by Mazure and Pouyssegur) [81]. Moreover, HIF-1 promotes the pro-autophagic signaling pathways in adjacent tumor stroma, which not only provides cancer cells with necessary chemical building blocks but also renders them less susceptible to apoptosis [82].

p53 involvement in autophagy appears to rely on two contradictory functions. On one hand, p53 facilitates autophagy by inducing expression of a damage-regulated autophagy modulator (DRAM) [83], sestrin 1, sestrin 2, AMP-dependent kinase (AMPK) [84] and/or inhibiting mTOR pathway [85]. On the other, Tasdemir et al. [86] postulate that cytoplasmic fraction of p53 can repress autophagy through a transcription-independent effect and that p53 inactivation enhances this process. On the contrary, Naves et al. [87] found that neuroblastoma cells with the mutated p53 undergo autophagy when exposed to hypoxia mimetic CoCl_2_, but this pathway is activated when p53 localizes to the nucleus. The studies quoted above show that the ‘self-digestion’ is another example of the mutual communication between HIF-1 and p53 in regulation of the tumor cells survival.

Recent developments in the field of senescence, a process leading to elimination of damaged cells from the growing population and subsequently preventing cancer occurrence, reveal a dual role for hypoxia. Leontieva et al. [88] found that hypoxia inhibits a conversion from the reversible cell cycle arrest to senescence (known as geroconversion), nutlin-induced senescence and mTOR activity. Additionally, in marrow-derived mesenchymal stem cells (MSCs) hypoxia promotes proliferation [89] and causes downregulation of p21^WAF1^ expression in a HIF-1α-dependent manner [90]. On the other hand, many of HIF-1- regulated genes are associated with the senescence induction, including plasminogen activator inhibitor (PAI1), cell cycle regulators, glycolytic enzymes and secreted molecules (see review by Welford et al.) [91]. The classic model of senescence shows that hyperoxia can induce senescence through reactive oxygen species (ROS). In accordance, senescence is inhibited under low oxygen conditions simply due to decreased production of the mitochondrial ROS [92]. Interestingly, recent report indicates that overexpression of caveolin-1 in the cancer-associated fibroblasts causes induction of their senescence and supports tumor growth due to HIF-1α stabilization by ROS increase [93]. In addition, VHL loss induces senescence in an oxygen-dependent manner by increasing the level of p27, which regulates cell cycle. However, these effects do not rely on HIF-1α or HIF-2α activity [94]. p53 involvement in senescence has been intensively studied till nowadays and recent achievements in that field have been profoundly reviewed [95-97]. It is noteworthy that p53 induction together with the prolonged p21^WAF1^ overexpression can suppress senescence in favor of quiescence [98].

**Figure 2:**
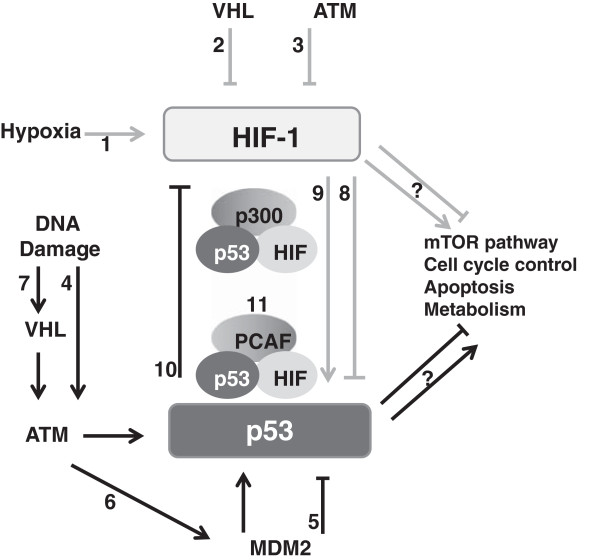
**Schematic characterization of mutual relations between HIF-1 and p53 pathways under different stress conditions.** 1. Activation of hypoxia-inducible factor (HIF-1) during hypoxia; 2. Suppression of HIF-1 by von Hippel Lindau protein (VHL) during normoxia; 3. Downregulation of HIF-1 expression and protein biosynthesis by ataxia-telangiectasia mutated protein (ATM); 4. Stabilization and phosphorylation of p53 triggered by ATM in response to DNA damage; 5. Murine double minute 2 (MDM2)-dependent ubiqutination of p53; 6. Activation of p53 dependent on ATM-mediated phosphorylation of MDM2; 7. Positive regulation of p53 during DNA damage by nucleating ATM mediated by VHL; 8. Downregulation of p53 by HIF-1 under mild hypoxia; 9. Activation of p53 by HIF-1 under severe or/and prolonged hypoxia; 10. Suppression of HIF-1 by p53 under anoxia; 11. Competition of p53 and HIF-1 for binding the cofactors p300 and PCAF during hypoxia.

Importantly, the cross-talk between p53 and HIF-1 can be observed at the level of their regulation, within a complex molecular loop which involves both factors (Figure 2). As mentioned before, ATM mediates a DNA double strand break signaling and repair via phosphorylation of p53. Ousset et al. [99] used various cellular models where ATM was disrupted and demonstrated that the absence of ATM increases expression of both subunits of HIF-1 as well as protein biosynthesis, through oxidative stress. However, ATM is also responsible for the phosphorylation of HIF-1 on Ser-696, which causes a downregulation of mTORC1 signaling that regulates a translational efficiency [100]. Not only hypoxia suppresses the mTOR pathway; p53 in response to stress also negatively regulates mTORC1 by inducing the expression of a plethora of target genes in the IGF-1/AKT and mTOR pathways. This intrinsic regulation was reviewed previously [29].

Another crosstalk between HIF-1 and p53 is observed on the level of trans-activation. During hypoxia, these transcription factors compete for the binding to the CH1 domain of p300 cofactor [101]. Furthermore, it was found that another cofactor, p300/CBP Associated Factor (PCAF) is involved in this regulatory mechanism. A study carried out by Xenaki et al. [102] focused on the expression of the pro-apoptotic p53 target BID and revealed a molecular mechanism underlying the regulation of p53 transcriptional activity in hypoxia. They have shown that hypoxia not only enables preferential direction of p53 to the promoter of p21^WAF1^ cell cycle arrester via PCAF, but also decreases PCAF-dependent acetylation of p53, which disrupts binding to its pro-apoptotic targets. They found that PCAF is also a HIF-1 cofactor involved in HIF-1- mediated apoptosis, whereas PCAF histone acetyltransferase (HAT) activity regulates transcriptional selectivity.

Additional convergences are visible on the level of regulation of these two transcription factors by VHL, which as mentioned above, is a well-documented ubiquitin-dependent executer of HIF-1 degradation [2,103]. However, it was also reported that VHL positively regulates p53 activity, preceded by DNA damage, via nucleating ATM and histone acetyltransferase to p53. It also influences cell cycle arrest and apoptosis triggered by p53 due to upgrading the p53-p300 interaction and p53 acetylation [103]. Moreover, ATF3 links the molecular pathways of HIF-1 and p53 in response to DNA-damage, where both transcription factors are over-represented, which can be explained by the suggestion that ATF3 synergizes with these transcription factors to modulate their target gene expression [39]. Recently, FIH was added to an even more complicated network in which p53 and HIF-1 are involved: FIH silencing in colon adenocarcinomas and melanoma cells greatly abolishes cell proliferation and, more importantly, increases both p53 and p21^WAF1^ protein levels [104]. These results support the role of FIH in the suppression of the p53-p21^WAF1^ axis.

## Impact of the p53 and HIF-1 interplay on cancer progression

Despite the fact that p53 is known to prevent mutations which cause genome instability and can lead to carcinogenesis, it represents one of the most frequently mutated genes in solid tumors [45]. Conformational changes related to missense mutations in the DNA-binding domain disrupt p53 transcriptional activity resulting in impaired ability of p53 to regulate the cellular response to hypoxia in an effective way [105,106]. It was also established that low oxygen pressure selects cells carrying p53 mutation and due to that contributes to metastatic potential and diminished apoptosis [46,107]. Interestingly, Gogna et al. [60] using *in-vivo* electron paramagnetic resonance oximetry 3D imaging found that conformationally mutated p53 appears in tumor hypoxic core and that its conformation is oxygen-dependent.

Furthermore, not only p53 mutations act in favor of cancer progression. Also hypoxia correlates with more aggressive tumor phenotypes and poor responses to therapy [108]. This mainly involves stabilization of HIF-1 and overexpression of its target genes [109]. For instance, expression of a HIF-1 target CA IX has been investigated in various types of cancers, including breast, colorectal, pancreatic etc. [110-112]. In these reports overexpression of this hypoxic marker was associated with poorer patient survival, less differentiated tumors of higher grade and worse response to therapy. Similar effects were described for VEGF in lung and gastric cancers [20,113]. Interestingly, high expression of HIF hydroxylases, which negatively regulate HIF-1 and are themselves regulated by hypoxia were postulated as poor prognostic factors in non small cell type lung cancers [114], whereas their inhibition reduced survival of glioblastoma cells [115]. Concurrent overexpression of both HIF-1 and p53 was found in many cancers as well [116]. An i*n vivo* study, based on an experimental model of chick embryo chorioallantoic membrane, revealed that HIF-1α increases invasiveness of human small cell lung carcinoma via promoting angiogenesis not only due to overexpression of VEGF but also due to secretion of pro-inflammatory factors [20]. Moreover, Khromova et al. [117] found that accelerated growth of cancer cells is associated with p53 mutations and caused by ROS-mediated activation of the HIF-1/VEGF-A pathway, which links both factors with neovascularization. In a large cohort of colorectal cancers, HIF-1α but not HIF-2α was shown to have an important negative prognostic role in cancer aggressiveness and overall survival of patients [118]. Contradictory to that, Cleven et al. [110] suggested that in the stroma of these tumors HIF-2α and CA IX serve as poor prognostic factors in tumors expressing wild-type p53 compared with tumors with mutant form. Regarding p53, some studies join its expression with patient survival [119] another with invasion depth [120] and poor differentiation [111] or worse distant survival [121]. Moreover, another report indicates no significant survival difference between wild-type and mutant p53 [110]. This leaves an open question on how hypoxia selects for mutated p53 and thereby impacts on patient outcome.

Hypoxia causes resistance to commonly used anti-cancer agents either due to downregulation of genes that are drug targets or because oxygen deprivation abrogates activity of the drugs. Chemotherapeutics of the first choice (doxorubicin, etoposide, cisplatin) cause DNA damage and therefore activate p53 to conduct apoptosis. HIF-1 by modulating expression of its target genes, render the cells less prone to treatment, although this effect is cell type-dependent [55]. Insensitivity can be HIF-1 independent as well, but relies on p53 suppression [122]. Moreover, hypoxic cells divide less rapidly and are localized further from functional blood vessels. Due to that, drugs are unable to reach poorly oxygenated areas and work less efficiently than in highly proliferating cells [123].

Last but not least, overexpression of P-glycoprotein (Pgp), a member of ATP-binding cassette (ABC) protein superfamily has been reported to cause multidrug resistance (MDR) of tumors [124,125]. Other studies elucidated that increase in Pgp abundance is due to transactivation by HIF-1 recruited to the MDR-1 gene in MCF-7 spheroids and hypoxic cells. Importantly, both MCF-7 spheroids and hypoxic cells show lower susceptibility to doxorubicin treatment and reduced accumulation of drugs [126].

## Conclusions

It is well known that hypoxia and genome instability are intrinsic tumor characteristics, which influence cancer progression and hence patient outcome. This report describes mutual relations between p53 and HIF-1 as mediators of adaptation to diverse cellular stresses, including DNA damage and hypoxia. Although they share many similarities, they can either act in parallel or compete with each other in regulation of diverse molecular pathways. These discrepancies have been extensively studied, but there are still many gaps in understanding what triggers pro-survival or lethal activity of these transcription factors. This work highlights the importance of further investigation of this loop as the data mentioned above indicate that it involves both positive and negative regulators as well as epigenetic mechanisms. This knowledge is indispensable not only for proper patient treatment, which as reported here can be influenced by both cancer cell type and tumor environment, but also for development of new drugs targeting p53 and/or HIF-1 pathways.

## Abbreviations

ABC: ATP-binding cassette protein superfamily; ATM: Ataxia telangiectasia mutated protein; BNIP3: BCL2/adenovirus E1B 19kDa-interacting protein 3; BNIP3L: BCL2/Adenovirus E1B 19kDa Interacting Protein 3-Like; CA IX: Carbonic anhydrase IX; DRAM: Damage-regulated autophagy modulator; FIH: The factor inhibiting HIF; HIF: hypoxia inducible factor; HIPK2: Homeodomein-interacting protein kinase-2; HREs: Hypoxia responsive elements; JAK2: Janus Activated Kinase; MAPK: Mitogen-activated protein kinase; MDM2: Murine double minute 2; MDR: Multidrug resistance; MSCs: Marrow-derived mesenchymal stem cells; mTOR: mammalian target of rapamycin; NO: Nitric oxide; PAI1: Plasminogen activator inhibitor; PCAF: p300/CBP Associated Factor; PHDs: Prolyl hydroxylase family; Pgp: P-glycoprotein; pVHL: Von Hippel-Lindau tumor suppressor protein; ROS: Reactive oxygen species; RPA70: 70 kDa subunit of replication protein A; SCO2: Cytochrome c oxidase 2; USP19: Ubiquitin-specific protease 19; Wip1: Wild-type p53-induced phosphatase 1; VEGF: Vascular endothelial growth factor.

## Competing interests

The authors declare they have no competing interests.

## Authors’ contributions

JO reviewed the literature, and wrote and edited the manuscript. SP contributed to study conception and critically revised the paper. BV critically revised the paper. RH contributed to study conception, revised and finalized the manuscript. All authors read and approved the final manuscript.
